# The complete chloroplast genome of Tibetan medicine *Gentianopsis paludosa*

**DOI:** 10.1080/23802359.2020.1714494

**Published:** 2020-01-20

**Authors:** Lucun Yang, Feng Xiong, Yuanming Xiao, Jingjing Li, Chen Chen, Changbin Li, Lingling Wang, Guoying Zhou

**Affiliations:** a Northwest Institute of Plateau Biology, Chinese Academy of Sciences, Xining, China;; b Qinghai Key Laboratory of Qinghai-Tibet Plateau Biological Resources, Chinese Academy of Sciences, Xining, China;; c Key Laboratory of Tibetan Medicine Research, Chinese Academy of Sciences, Xining, China;; d Research Center of Biological Resources in Qinghai-Tibet Plateau, University of Chinese Academy of Sciences, Beijing, China;; e College of Life Science, Qinghai Normal University, Xining, China

**Keywords:** *Gentianopsis paludosa*, Complete chloroplast, phylogeny

## Abstract

*Gentianopsis paludosa* (Mum.) Ma is an important species in Tibetan folk medicine, but its wild populations are shrinking roughly due to the increasing demand for it. *Gentianopsis paludosa* is presently at risk of over-exploitation, so it needs urgent conservation. Here, we report the complete sequence of the chloroplast genome of *G. paludosa*. The genome was 51,121 bp in length with 129 genes comprising 84 protein-coding genes, 37 tRNA genes, and eight rRNA genes. The overall GC content of *G. paludosa* chloroplast genome was is 36.67%. The phylogenomic analysis suggested that *G. paludosa* forms a clade with species in *Halenia* and *Swertia*, indicating that the *G. paludosa* is more closely related to *Halenia* and *Swertia* than that of *Gentiana*.

*Gentianopsis paludosa* (Mum.) Ma, belonging to the family Gentianaceae, is an important species in Tibetan folk medicine. Tibetan people called it jiadi, jihedou, etc. (Lu et al. 2016). Its natural range is on the Qinghai-Tibetan Plateau of China with a high altitude (2500–4500 m) (Liu 1996). The whole herb of *G. paludosa* is widely used for treatment of conjunctivitis, hepatitis, nephritis, gastroenteritis, dyspepsia, fever, influenza, and diarrhea due to the high levels of oleanolic acid, gentiopicrin, and swertiamarin contained in *G. paludosa* (Guo 1987; Tu et al. 2005; Cai et al. 2009). Moreover, researchers found that *G. paludosa* had anti-diarrhea and antibacterial activity (Wang et al. 2005), which promoted the increasing demand for it. As a result of the increasing demand for it, *G. paludosa* is presently at risk of over-exploitation (Liu 1996). To date, previous studies of *G. paludosa* have mainly focused on chemical composition (Wang et al. 2005; Vaidya et al. 2009), genetic diversity (Yu et al. 2012), pharmacodynamics (Lu et al. 2017), and others (Xue and Li 2011). Thus, it is useful to know more genetic information about *G. paludosa* to carry out conservation, genetic improvement, and sustainable management for this risk species.

We sampled the wild individual of *G. paludosa* from Xinping village (101°37.556′ E, 36°34.460′ N, 2510 m), Huangzhong country in Qinghai province of China (Voucher specimen: QHGC20190824, HNWP). Genome DNA was extracted using modified CTAB method, which was fragmented to construct paired-end (PE) libraries. Genome sequences were screened out and assembled with the SPAdes. And then, annotation was performed with DOGMA. The annotated genomic sequence had been submitted to GenBank with the accession number SRR10572767.

The complete chloroplast genome length of *G. paludosa* is 151,121 bp, consisting of two inverted repeats (IR, 25,224 bp) separated by a large single copy region (LSC 82,813 bp) and a small single copy region (SSC 17,860 bp). The overall CC content is 36.67%. The chloroplast genome includes a total of 129 functional genes including 84 protein-coding genes, 37 tRNA and eight rRNA. A total of 18 genes were duplicated in the IR regions including seven tRNA, four rRNA, and seven protein-coding genes. The genome organization, gene/intron content, and gene relative positions of the newly sequenced plastid genome were almost identical to other Gentianaceae species.

We used the complete chloroplast genomes of *G. paludosa* and 18 other species from Gentianaceae to reconstruct the Phylogenetic tree. And, *Carissa macrocarpa* (Apocynaceae) was used as an outgroup. Maximum-likelihood (ML) analysis demonstrated that *G. paludosa* formed a clade with species in *Halenia* and *Swertia* with high bootstrap values (Figure 1), indicating that the *G. paludosa* was more closely related to *Halenia* and *Swertia* than that of *Gentiana*.

**Figure 1.**
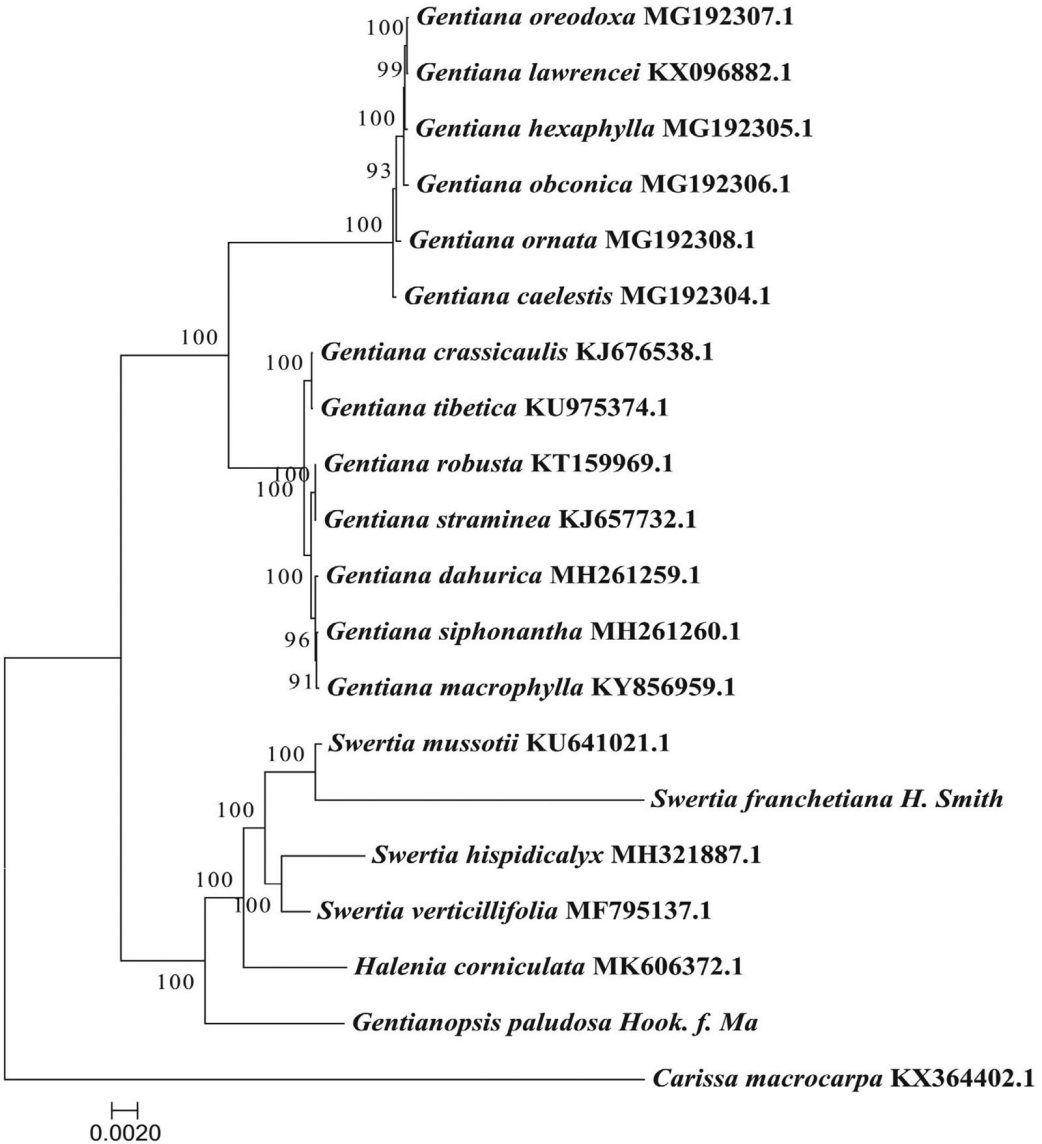
Maximum-likelihood phylogenetic tree based on 20 complete chloroplast genome sequences.
